# Ultra-early weaning alters growth performance, hematology parameters, and fecal microbiota in piglets with same genetic background

**DOI:** 10.3389/fmicb.2022.990905

**Published:** 2022-11-02

**Authors:** De Xin Dang, Cheng Ji Li, Shi Han Li, Xin Yan Fan, Weiguo Xu, Yan Cui, Desheng Li

**Affiliations:** ^1^College of Animal Science and Veterinary Medicine, Jinzhou Medical University, Jinzhou, China; ^2^Department of Animal Resource and Science, Dankook University, Cheonan, South Korea; ^3^Institute of Tissue Regeneration Engineering, Dankook University, Cheonan, South Korea; ^4^Department of Nanobiomedical Science and BK21 PLUS NBM Global Research Center for Regenerative Medicine, Dankook University, Cheonan, South Korea

**Keywords:** fecal microbiota, 16S, pig, immunity, ultra-early weaning, growth

## Abstract

Piglets with the same genetic background were used to investigate the effects of different lengths of suckling period on growth performance, hematology parameters, and fecal microbiota. All piglets were born by a sow (Landrace×Yorkshire). On day 14 postpartum, a total of 16 piglets [Duroc×(Landrace×Yorkshire)] with a similar initial body weight (2.48 ± 0.25 kg) were randomly assigned into two groups with four replicates per group, two pigs per replicate pen (one barrow and one gilt). On day 14 of age, experiment started, piglets from the first group were weaned (14W), whereas the others continued to receive milk until day 28 of age (28W). The experiment completed on day 70 of age, last 56 days. Growth performance parameters including body weight, average daily gain, feed intake, feed efficiency, and growth rate and hematology parameters including immunoglobulin A (IgA), immunoglobulin G (IgG), immunoglobulin M (IgM), albumin, globulin, and total protein were measured in this study. Additionally, a technique of 16S rRNA gene sequencing was used to analyze fecal microbiota for revealing how the changes in the lengths of suckling period on intestinal microbiota. We found that ultra-early weaning impaired growth performance of piglets, whose worse body weight, average daily gain, feed intake, feed efficiency, and growth rate were observed in 14W group at all measured timepoints in comparison with those in 28W group (*P* < 0.05). Moreover, higher contents of serum IgA (*P* = 0.028), IgG (*P* = 0.041), and IgM (*P* = 0.047), as well as lower contents of serum albumin (*P* = 0.002), albumin-to-globulin ratio (*P* = 0.003), and total protein (*P* = 0.004), were observed in 14W group in comparison with those in 28W group on day 28 of age, but not on day 70 of age. High-throughput pyrosequencing of 16S rRNA indicated that the intestinal microbiota richness in 14W group was lower than that in 28W group (*P* < 0.05); moreover, in comparison with 28W group at all sampling timepoints, fecal microbiota in 14W group showed more beneficial bacteria and fewer pathogenic bacteria (*P* < 0.05). Therefore, we considered that ultra-early weaning had positive effects on immune status and fecal microbiota composition in piglets, but negative effects on growth performance and fecal microbiota abundance.

## Introduction

Breast-feed provides a possibility for the vertical transmission of pathogens from sows to offspring (Smith et al., 2008). Additionally, with the prolonging of lactation period, the yield and quality of milk from sows failed to provide adequate nutrients to support the large growth potential of offspring (Nuntapaitoon, 2022). Therefore, consistently intaking low-quality milk will limit the growth of piglets. On the contrary, with the growth of piglets, sows are reluctant to suckle the piglets, and they will limit the piglets getting in touch with the udder through frequent posture adjustments and even attack them (EFSA Panel on Animal Health and Welfare, 2022). Therefore, shortening the suckling period seems to have positive effects on the growth of piglets and the welfare of sows. Some studies reported that shortening the suckling period improved the reproductive performance and body conditions of sows (Spencer et al., 2003; Holman et al., 2021), as well as increased the economic value of piglets (Main et al., 2004, 2005). However, some studies reported that shortening suckling period negatively affected the growth performance (Ming et al., 2021), intestinal health (Cao et al., 2022), immune status (Tao et al., 2016), antioxidant capacity (Buchet et al., 2017), nutrient digestibility (Ming et al., 2021), and survival rate (Huting et al., 2019), as well as led to diarrhea, prolonged the required days to reach marketing weight, and increased feed cost (Smith et al., 2008; Massacci et al., 2018; Faccin et al., 2020a,b).

Recently, the relationship between gut microbes and productive performance of animals has received unprecedented attention (Isaacson and Kim, 2012). Studies on the effects of ultra-early weaning on intestinal microbiota of pigs are still limited.

Moreover, genetic background plays a key role in affecting the individual differences of animals (Champy et al., 2008). The same genetic background means the minimization of individual differences. Therefore, this study investigated the effects of ultra-early weaning on growth performance, hematology parameters, and fecal microbiota in piglets. According to the recommendation of Mabry et al. (1996) and Xue et al. (1997), piglets were weaned on day 14 or 28 of age in this study.

We hypothesized that shortening suckling period had negative effects on the growth performance and hematology parameters, moreover, led to the disorder in intestinal microbiota, and manifested in the increase of pathogenic bacteria and the decrease of beneficial bacteria. The objective of this study was to evaluate the effects of ultra-early weaning on growth performance, hematology parameters, and fecal microbiota in piglets.

## Materials and methods

### Experimental design, animals, and housing

A total of 16 14-day-old piglets [Duroc×(Landrace × Yorkshire)] with a similar initial body weight (2.48 ± 0.25 kg) were selected from the same sow (Landrace×Yorkshire) for ensuring the same genetic background. All piglets were randomly assigned into two groups with four replicates, two piglets per replicate pen (one barrow and one gilt). The experimental factor was the lengths of suckling period, of which piglets from the first group were weaned on day 14 of age (14W) and others were weaned on day 28 of age (28W). The experiment lasted to day 70 of age (56 days). In the group of 14W, piglets received creep feed during days 14 to 28 of age. On day 29 of age, all piglets were given the same feed, which was formulated to meet the recommendation of the National Research Council [NRC] (2012) and provided in a mashed form (Table 1). Experimental protocol (no. JMU00211232) and the process were approved and supervised by the Animal Care and Use Committee of Jinzhou Medical University (Jinzhou, China). The care and the treatment of the sows were according to the animal welfare legislation (Federation of Animal Science Societies, 2010).

**TABLE 1:** Composition and nutrient levels of the experimental basal diet during post-weaning period (%, as-fed basis).

Ingredients, %	Days 14–28 of age^1^	Days 29–70 of age^2^
Corn	35.92	48.09
Puffed corn	18.00	15.00
Soybean meal	12.00	18.50
Fermented soybean meal	12.00	6.00
Whey protein	10.00	5.00
Fish meal	4.00	3.00
Corn starch	–	0.20
Spray-dried porcine plasma	3.00	–
Soy oil	2.20	1.08
Monocalcium phosphate	0.80	0.66
Limestone	0.60	0.90
Mineral and vitamin mixture^3^	0.40	–
Mineral and vitamin mixture^4^	–	0.50
Lysine	0.40	0.39
Salt	0.30	0.30
Threonine	0.15	0.16
Choline	0.10	–
Methionine	0.12	0.20
Tryptophan	0.01	0.02
Total	100.00	100.00
**Analyzed composition,%**		
Crude protein	20.77	18.88
Metabolizable energy, MJ/kg	14.83	14.61
Lysine	1.54	1.24
Threonine	1.01	0.73
Calcium	0.81	0.70
Methionine	0.45	0.36
Available phosphorus	0.37	0.34
Tryptophan	0.26	0.20
Crude fat	4.94	4.03
Crude fiber	5.56	5.32
Ash	2.18	2.47

^1^Dietary composition of piglets weaned on day 14 of age (ultra-early weaning group).

^2^Dietary composition of piglets in all groups.

^3^Provided per kg of complete diet: Zn 100 mg; Mn 4 mg; Fe 100 mg; Cu 100 mg; I 0.3 mg; Se 0.3 mg; vitamin A 14000 IU; vitamin D_3_ 4000 IU; vitamin E 4.7 mg; vitamin B_1_ 4 mg; vitamin B_2_ 10 mg; vitamin B_6_ 6 mg; vitamin B_12_ 0.04 mg; niacin 40 mg; pantothenic acid 20 mg; folic acid 2 mg; biotin 0.16 mg.

^4^Provided per kg of complete diet: Zn 80 mg; Mn 4 mg; Fe 100 mg; Cu 200 mg; I 0.14 mg; Se 0.25 mg; choline chloride 400 mg; vitamin A 10500 IU; vitamin D_3_ 3000 IU; vitamin E 22.51 IU; vitamin K_3_ 3 mg; vitamin B_1_ 3 mg; vitamin B_2_ 7.5 mg; vitamin B_6_ 4.5 mg; vitamin B_12_ 0.03 mg; niacin 30 mg; pantothenic acid 15 mg; folic acid 1.5 mg; biotin 0.12 mg.

Piglets did not receive creep feed during the suckling period. On the third after birth, piglets were subjected to routine management practices and received 1 ml of iron dextran (50 mg/kg). Male piglets were castrated.

All piglets were housed in an environmentally controlled nursery barn. The ambient temperature within the room was maintained at 30°C until day 35 of age and reduced by 1°C per week subsequently. The humidity was around 60%. Piglets had free access to feed and water.

### Sampling and measurements

#### Growth performance

All piglets were weighed on days 14, 28, and 70 of age to calculate the average daily gain (ADG) and growth rate. Daily feed intake was recorded to measure the average daily feed intake (ADFI) based on the pen. Feed efficiency was calculated according to the values of ADG and ADFI.

#### Hematology parameters

All piglets were used for collecting blood *via* jugular venipuncture on days 28 and 70 of age. Blood samples (5 mL) were collected into vacuum tubes without anticoagulants (Becton Dickinson Vacutainer Systems, Franklin Lakes, NJ, USA). After collection, the blood samples were centrifuged (3500 × *g*) for 10 min at 4°C to extract the serum and then stored at −20°C until analysis. The contents of IgA, IgG, and IgM were measured by specific ELISA kit (Meimian Industrial Ltd., Co., Jiangsu, China). Additionally, the concentrations of albumin, globulin, and total protein were measured by a Beckman-CX4 automatic biochemical analyzer (Beckman Coulter, Inc., Brea, CA, USA).

#### Fecal microbiota analysis by 16S rRNA gene sequencing

Fresh stool samples were taken from 16 piglets. The specimens were kept in ice boxes until they arrived at the laboratory. A Magnetic Soil and Stool DNA Kit (cat# DP712, Tiangen Biotech Co., Ltd., Beijing, China) was used for extracting total DNA from 16 fecal samples (0.5 g). The concentration and purity of the extracted DNA were determined using a Qubit 2.0 spectrophotometer (Invitrogen, Carlsbad, CA, USA) and 1% (w/v) agarose gel electrophoresis. The quality of DNA was judged according to the results of agarose gel electrophoresis, and the result of “A” was considered high-quality DNA. The DNA samples were diluted with sterile water to a concentration of 1 ng/μL and stored at −20°C before analysis. Then, the V3–V4 hypervariable regions of the bacterial 16S rRNA gene were amplified with specific full-length universal forward (5′-ACTCCTACGGGAGGCAGCAG-3′) and reverse (5′-GGACTACHVGGGTWTCTAAT- 3′) primers. PCRs were performed in triplicate with each 20 μL reaction mixture containing 4 μL of 5 × FastPfu buffer, 2 μL of 2.5 mM dNTPs, 0.8 μL of each primer (5 μM), 0.4 μL FastPfu polymerase, and 10 ng of template DNA. The PCR conditions were 95°C for 3 min; 95°C for 30 s, 55°C for 30 s, and 72°C for 45 s, repeat for 27 cycles; and 72°C for 10 min. Subsequently, a Qiagen Gel Extraction Kit (cat# 28706, Qiagen, Germany) was used to further purify the PCR products. Simultaneously, the purity of the PCR mixture was evaluated using a Qubit 2.0 dsDNA HS Assay Kit (cat# Q32854, Invitrogen). The 16S rRNA gene sequencing was performed to analyze the fecal microbial community structures using the NovaSeq 6000 platform (Illumina, San Diego, CA, USA) in Novogene Bioinformatics Co., Ltd. (Tianjin, China).

Raw data were obtained by cutting low-quality reads using Cutadapt software version 1.9.1. Chimeric sequences were trimmed by alignment and detection. High-quality reads were clustered into operational taxonomic units (OTUs) at 97% sequence identity using Uparse v7.0.1001. The taxonomic assignment of the representative sequences was performed using QIIME v1.9.1. A rarefaction curve was plotted for each sample using R software (version 1.9.1) to determine the suitable sequencing depth that covers the extent of microbial diversity. The number of observed OTUs was used to calculate alpha-diversity, including observed species, Chao1, Ace, Shannon, and Simpson diversity indices, and beta-diversity, including Bray–Curtis and unweighted UniFrac. The calculation of construction of weighted pair-group method with arithmetic mean (UPGMA) trees was done using QIIME and R package software.

## Statistical analysis

All data were examined for normality by Shapiro–Wilk test and QQ plots. Replicate served as the experimental unit. Student’s *t*-test was used to analyze the data of hematology parameters as well as the alpha-diversity and beta-diversity from fecal microbiota by SPSS software (version 21.0). The results were presented as the means ± standard deviation. Spearman’s analysis was used to evaluate the correlations between fecal microbiota and immunology parameters. Moreover, the growth performance parameters were analyzed by a MIXED procedure for repeated measurements at different sampling timepoints in which the statistical model accounted for the main effects of treatment, time, and their interaction. Tukey’s *post-hoc* test was used to separate means among treatments. Variability in the data of growth performance was expressed as the standard error of means. A probability value below 0.05 was taken to denote statistical significance.

## Results

Ultra-early weaning had negative effects on the growth performance of piglets, of which piglets in the group of 14W had lower body weight on days 28 (*P* = 0.027) and 70 (*P* = 0.001) of age, ADG during days 14–28 (*P* = 0.013), days 29–70 (*P* = 0.001), and days 14–70 (*P* = 0.001) of age, growth rate during days 14–28 (*P* = 0.020), days 29–70 (*P* = 0.048), and days 14–70 (*P* = 0.006) of age, ADFI during days 29–70 of age (*P* = 0.001), and feed efficiency during days 29–70 of age (*P* = 0.028) in comparison with those in the group of 28W (Table 2). Additionally, there was a significant time effect for body weight (*P* < 0.001), ADG (*P* < 0.001), and growth rate (*P* < 0.001). The mean value of body weight (*P* < 0.001), ADG (*P* < 0.001), and growth rate (*P* < 0.001) from the group of 14W was lower than that from the group of 28W. Interactions between time and treatment were also observed for body weight (*P* < 0.001), ADG (*P* = 0.043), and growth rate (*P* < 0.001).

**TABLE 2:** Effects of ultra-early weaning on the growth performance in post-weaning piglets measured at different timepoints.

Items	28W^1^	14W^2^	SEM^3^	*P*-value		
				Time	Treatment	Time × treatment
**Body weight, kg**						
Day 14 of age	2.53	2.43	0.108		0.516	
Day 28 of age	6.09	5.29	0.205		0.027	
Day 70 of age	22.56	18.03	0.551		0.001	
Mean	10.39	8.58	0.164	<0.001	<0.001	<0.001
**Average daily gain, g**						
Days 14–28 of age	237.25	190.42	10.025		0.013	
Days 29–70 of age	401.55	310.88	12.101		0.001	
Days 14–70 of age	357.54	278.62	9.179		0.001	
Mean	332.12	259.97	5.020	<0.001	<0.001	0.043
**Average daily feed intake, g**						
Days 14–28 of age		232.09				
Days 29–70 of age	600.89	508.77	12.758		0.001	
Days 14–70 of age		370.43				
**Feed efficiency^4^**						
Days 14–28 of age		0.83				
Days 29–70 of age	0.67	0.61	0.017		0.028	
Days 14–70 of age		0.75				
**Growth rate^5^**						
Days 14–28 of age	2.42	2.18	0.075		0.020	
Days 29–70 of age	3.73	3.42	0.121		0.048	
Days 14–70 of age	8.99	7.44	0.337		0.006	
Mean	5.044	4.345	0.095	<0.001	<0.001	<0.001

^1^Piglets weaned on day 28 of age.

^2^Piglets weaned on day 14 of age.

^3^Standard error of means.

^4^Feed efficiency was calculated as the ratio of feed to gain.

^5^Growth rate was calculated as the ratio of final body weight to initial body weight.

On day 28 of age, piglets from the group of 14W had higher serum IgA (*P* = 0.028), IgG (*P* = 0.041), and IgM (*P* = 0.047) concentrations and lower serum albumin (*P* = 0.002) and total protein (*P* = 0.004) concentrations as well as albumin-to-globulin ratio (*P* = 0.003) than those from the group of 28W (Table 3).

**TABLE 3:** Effects of ultra-early weaning on the serum biochemical indicators in post-weaning piglets.

Items	28W^1^	14W^2^	*P*-value
**Immunoglobulin A, mg/L**			
Day 28 of age	684.44 ± 37.36	825.71 ± 49.83	0.028
Day 70 of age	768.57 ± 74.10	649.52 ± 54.74	0.139
**Immunoglobulin G, g/L**			
Day 28 of age	19.92 ± 1.09	22.97 ± 0.88	0.041
Day 70 of age	19.09 ± 0.48	17.84 ± 0.66	0.101
**Immunoglobulin M, g/L**			
Day 28 of age	14.40 ± 0.90	17.04 ± 1.04	0.047
Day 70 of age	13.76 ± 0.77	14.48 ± 0.28	0.282
**Albumin, g/L**			
Day 28 of age	36.23 ± 4.52	23.35 ± 1.76	0.002
Day 70 of age	32.30 ± 1.50	30.43 ± 2.29	0.220
**Globulin, g/L**			
Day 28 of age	16.55 ± 0.81	16.58 ± 1.14	0.973
Day 70 of age	18.33 ± 0.86	18.38 ± 1.76	0.961
**Albumin to globulin ratio**			
Day 28 of age	2.19 ± 0.30	1.41 ± 0.11	0.003
Day 70 of age	1.76 ± 0.11	1.67 ± 0.21	0.433
**Total protein, g/L**			
Day 28 of age	52.78 ± 4.16	40.18 ± 3.56	0.004
Day 70 of age	50.63 ± 1.86	48.80 ± 3.00	0.341

The results were presented as mean ± standard deviation.

^1^Piglets weaned on day 28 of age.

^2^Piglets weaned on day 14 of age.

As observed in Table 4, the results indicated that piglets from the group of 14W had lower Chao1 index (*P* = 0.002) and Ace index (*P* = 0.004) than those from 28W group on day 28 of age. However, the Shannon and Simpson diversity did not differ among the groups at different sampling timepoints. In addition, observed species (*P* = 0.008) in the group of 14W was lower than that in the group of 28W on day 28 of age (Table 4).

**TABLE 4:** Summary of next generation sequencing data and effects of ultra-early weaning on diversity and abundance indexes at each sampling time in post-weaning piglets.

Alpha diversity indexes	28W^1^	14W^2^	*P*-value
**Observed species**			
Day 28 of age	754.33 ± 107.53	596.67 ± 45.56	0.008
Day 70 of age	759.33 ± 134.77	666.33 ± 99.52	0.204
**Shannon index**			
Day 28 of age	5.56 ± 0.61	5.01 ± 0.57	0.139
Day 70 of age	6.17 ± 0.20	6.26 ± 0.12	0.365
**Simpson index**			
Day 28 of age	0.92 ± 0.04	0.88 ± 0.06	0.155
Day 70 of age	0.95 ± 0.01	0.97 ± 0.01	0.130
**Chao1 index**			
Day 28 of age	919.79 ± 140.35	653.51 ± 74.67	0.002
Day 70 of age	768.13 ± 140.16	679.52 ± 101.57	0.238
**ACE index**			
Day 28 of age	916.83 ± 145.99	673.47 ± 72.69	0.004
Day 70 of age	787.97 ± 148.60	699.02 ± 104.76	0.258

The results were presented as mean ± standard deviation.

^1^Piglets weaned on day 28 of age.

^2^Piglets weaned on day 14 of age.

Spearman’s correlations analysis between fecal microbiota and serum immunoglobulin parameters indicated that fecal microbiota in the level of genus were correlated with the serum immunoglobulin parameters (Table 5). Among them, on day 28 of age, the richness of *Prevotellaceae_NK3B31_group* was positively correlated with the concentrations of IgA (*P* = 0.005); the richness of *Prevotella* was positively correlated with the concentrations of IgM (*P* = 0.019); the richness of *Agathobacter* was positively correlated with the concentrations of IgA (*P* = 0.042); and the richness of *Prevotellaceae_UCG.003* was positively correlated with the concentrations of IgG (*P* = 0.019). On day 70 of age, the richness of *Prevotellaceae_UCG.003* (*P* = 0.042) and *Bacteroides* (*P* = 0.005) was negatively correlated with the concentrations of IgG.

**TABLE 5:** Spearman’s correlations analysis between fecal microbiota and serum immunoglobulin parameters.

Variables	IgA	IgG	IgM
**Day 28 of age**			
Prevotellaceae_NK3B31_group	0.943**	0.771	0.657
Christensenellaceae_R.7_group	–0.771	–0.600	–0.657
Prevotella	0.600	0.771	0.886**
Agathobacter	0.829*	0.657	0.486
Desulfovibrio	–0.771	–0.600	–0.657
Prevotellaceae_UCG.003	0.600	0.886**	0.714
Phascolarctobacterium	0.029	0.086	–0.029
**Day 70 of age**			
Prevotella	0.086	0.771	–0.406
Prevotellaceae_UCG.003	–0.543	−0.829*	0.406
Bacteroides	–0.371	−0.943**	0.464
Phascolarctobacterium	–0.486	–0.600	0.290
Solobacterium	–0.429	–1.000	0.232

IgA, immunoglobulin A; IgG, immunoglobulin G; IgM, immunoglobulin M. *P < 0.05; **P < 0.01.

**FIGURE 1:**
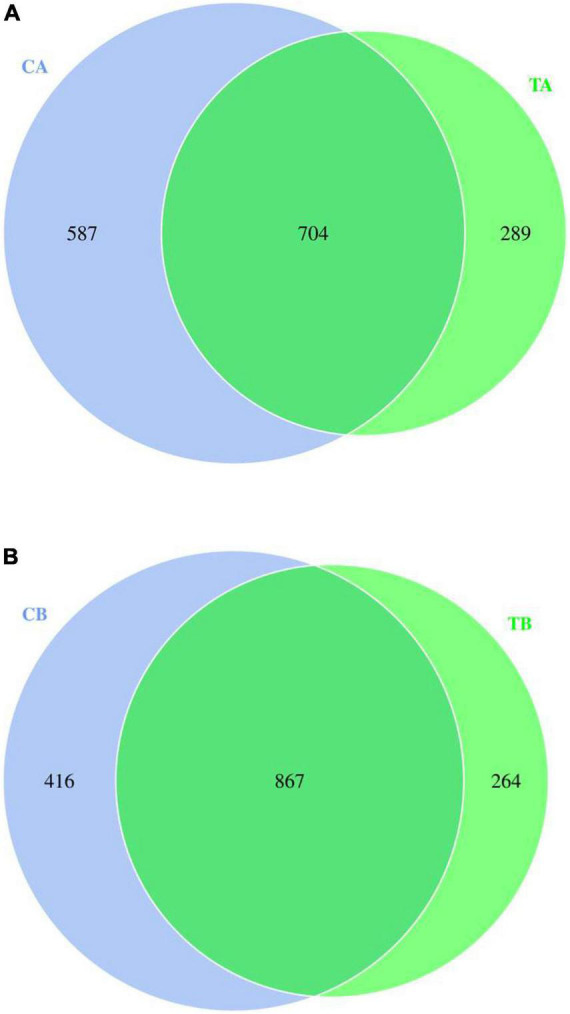
Venn graph for intestinal microbiota of piglets weaned on day 14 of age (14W) and day 28 of age (28W) at different sampling timepoints [day 28 of age **(A)**; day 70 of age **(B)**]. CA group was defined as the sample from piglets in 28W group at the timepoint of day 28 of age. TA group was defined as the sample from piglets in 14W group at the timepoint of day 28 of age **(A)**. CB group was defined as the sample from piglets in 28W group at the timepoint of day 70 of age. TB group was defined as the sample from piglets in 14W group at the timepoint of day 70 of age **(B)**.

The Venn diagram showed the distribution of bacterial unique OTUs among the groups based on the 16S rRNA gene sequencing analysis, and it visualized the distribution of shared and unique OTUs among the groups (the numbers within the Venn diagram represented the total number of OTUs in that community). Different colors represented different groups, and the number in the middle represented the number of OTUs shared by all groups. The distribution of fecal microbiota of piglets on day 28 of age is presented in Figure 1A, while that of piglets on day 70 of age is presented in Figure 1B. As shown in Figure 1A, 704 bacterial OTUs were shared among the groups, and the 587 unique OTUs in CA group (sample from piglets in 28W group on the timepoint of day 28 of age; 28W-28) and the 289 unique OTUs in TA group (sample from piglets in 14W group on the timepoint of day 28 of age; 14W-28) were observed. Additionally, as shown in Figure 1B, a total of 867 OTUs were identified by the Venn diagram as common to the treatments. The unique OTUs in CB group (sample from piglets in 28W group on the timepoint of day 70 of age; 28W-70) were 416, whereas those in TB group (sample from piglets in 14W group on the timepoint of day 70 of age; 14W-70) were 264.

**FIGURE 2:**
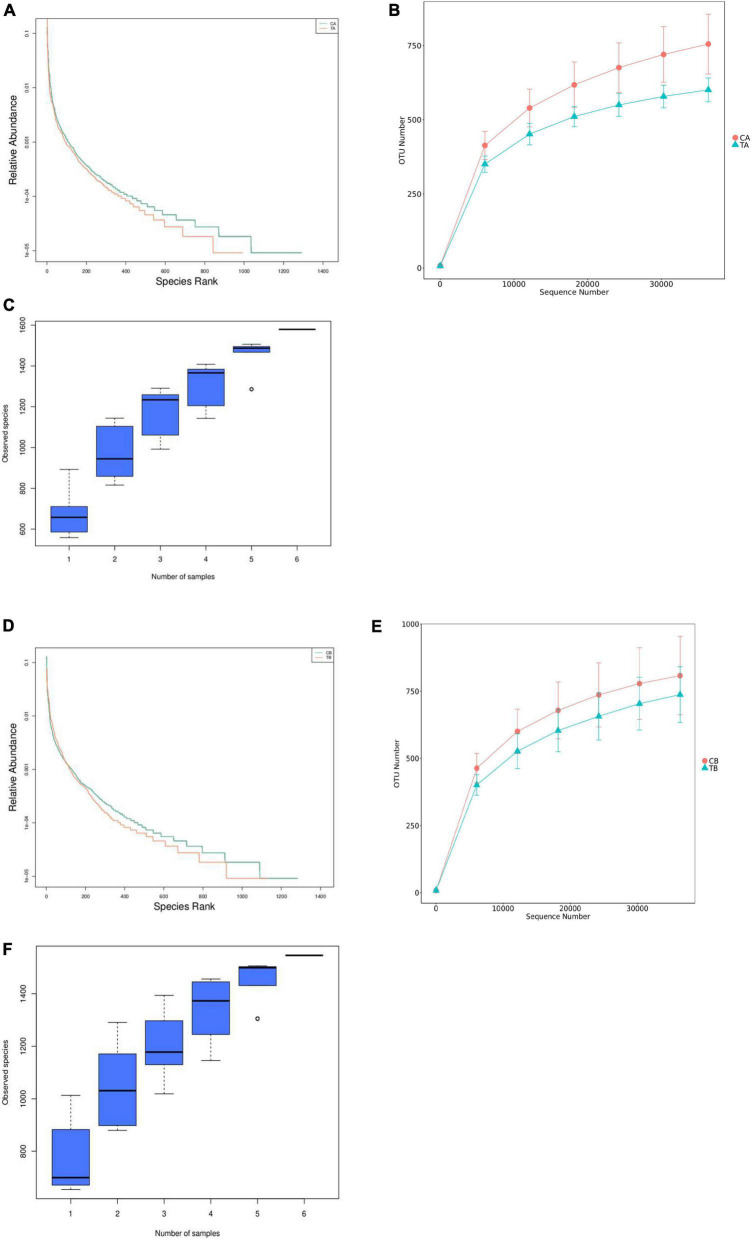
Rank abundance, rarefaction curves, and species accumulation boxplot for intestinal microbiota of piglets weaned on day 14 of age (14W) and day 28 of age (28W) at different sampling timepoints [day 28 of age **(A–C)**; day 70 of age **(D–F)**]. CA group was defined as the sample from piglets in 28W group at the timepoint of day 28 of age. TA group was defined as the sample from piglets in 14W group at the timepoint of day 28 of age **(A–C)**. CB group was defined as the sample from piglets in 28W group at the timepoint of day 70 of age **(D–F)**. TB group was defined as the sample from piglets in 14W group at the timepoint of day 70 of age.

The rank abundance (Figures 2A,D), rarefaction curves (Figures 2B,E), and species accumulation boxplot (Figures 2C,F) were adopted to access the richness of fecal bacteria community in each group and showed that the observed species gradually tend to be flat as the sample size increased, which indicated that the amount of data to be sequenced was reasonable, and the subsequent data and index analyses can be performed.

**FIGURE 3:**
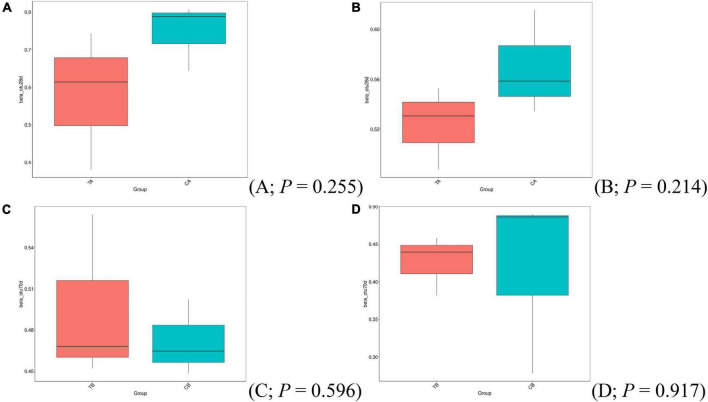
Beta-diversity analysis of Bray–Curtis **(A,C)** and unweighted UniFrac **(B,D)** using *t*-test for intestinal microbiota of piglets on day 14 of age (14W) and day 28 of age (28W) at different sampling timepoints [day 28 of age **(A,B)**; day 70 of age **(C,D)**]. CA group was defined as the sample from piglets in 28W group at the timepoint of day 28 of age. TA group was defined as the sample from piglets in 14W group at the timepoint of day 28 of age **(A,B)**. CB group was defined as the sample from piglets in 28W group at the timepoint of day 70 of age. TB group was defined as the sample from piglets in 14W group at the timepoint of day 70 of age **(C,D)**.

No statistical differences in beta-diversity indices (Bray–Curtis, Figures 3A,C; unweighted UniFrac, Figures 3B,D) have been observed based on the *t*-test at different sampling timepoints.

**FIGURE 4:**
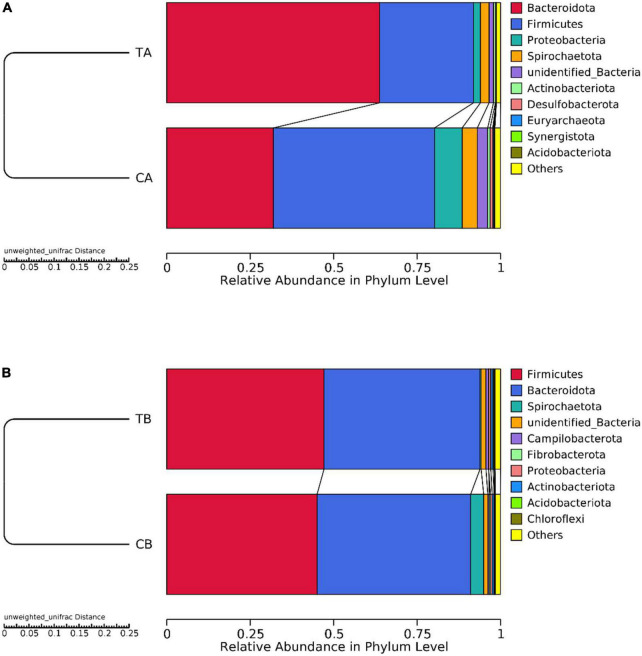
Taxonomic distribution at phylum level for intestinal microbiota of piglets weaned on day 14 of age (14W) and day 28 of age (28W) at different sampling timepoints [day 28 of age **(A)**; day 70 of age **(B)**]. The “others” represents the sum of the relative abundance except the top 10 in the figure. CA group was defined as the sample from piglets in 28W group at the timepoint of day 28 of age. TA group was defined as the sample from piglets in 14W group at the timepoint of day 28 of age **(A)**. CB group was defined as the sample from piglets in 28W group at the timepoint of day 70 of age. TB group was defined as the sample from piglets in 14W group at the timepoint of day 70 of age **(B)**.

The unweighted pair-group method with arithmetic mean cluster tree based on the unweighted UniFrac distance was used to examine the similarity between samples at phylum level. On day 28 of age (Figure 4A), *Bacteroidota* and *Firmicutes* were predominated and the remaining bacterial sequences were mainly assigned to *Proteobacteria*, *Spirochaetota*, *unidentified_Bacteria*, *Actinobacteriota*, *Desulfobacterota*, *Euryarchaeota*, *Synergistota*, and *Acidobacteriota*. On day 70 of age (Figure 4B), *Firmicutes* and *Bacteroidota* were predominant and the remaining bacterial sequences were mainly assigned to *Spirochaetota*, *unidentified_Bacteria*, *Campilobacterota*, *Fibrobacterota*, *Proteobacteria*, *Actinobacteriota*, *Acidobacteriota*, and *Chloroflexi*.

**FIGURE 5:**
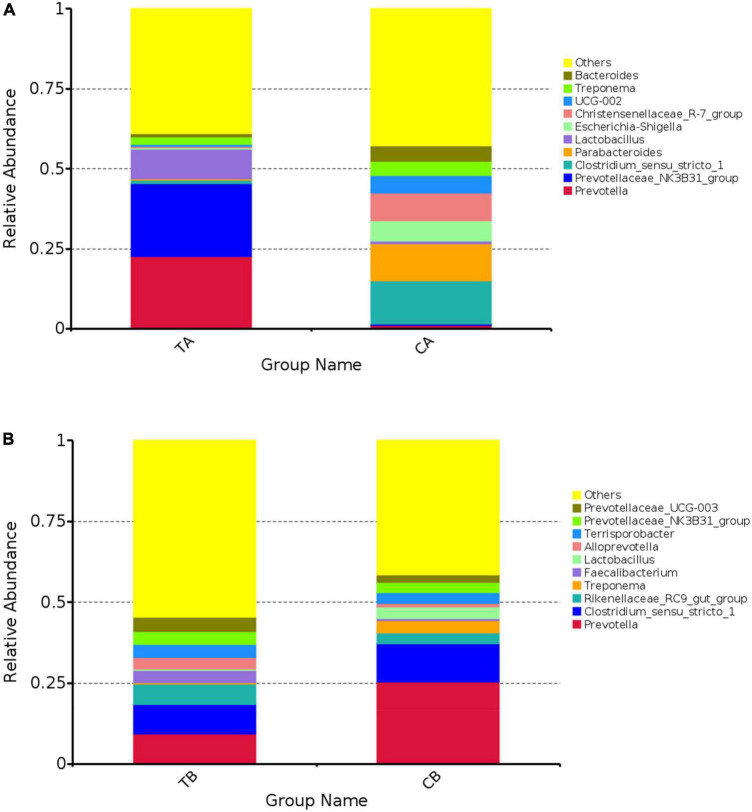
The 10 most abundant bacteria of gut microbiota at genus level for intestinal microbiota of piglets weaned on day 14 of age (14W) and day 28 of age (28W) at different sampling timepoints [day 28 of age **(A)**; day 70 of age **(B)**]. The horizontal axis is the groups. The vertical axis represents the relative abundance. The “others” represents the sum of the relative abundance except the top 10 in the figure. CA group was defined as the sample from piglets in 28W group at the timepoint of day 28 of age. TA group was defined as the sample from piglets in 14W group at the timepoint of day 28 of age **(A)**. CB group was defined as the sample from piglets in 28W group at the timepoint of day 70 of age. TB group was defined as the sample from piglets in 14W group at the timepoint of day 70 of age **(B)**.

The 10 most abundant bacteria of fecal microbiota at genus level are shown in Figure 5. As shown in Figure 5A, the predominant bacteria on day 28 of age were mainly involved in *Bacteroides*, *Treponema*, *UCG-002*, *Christensenellaceae_R-7_group*, *Escherichia–Shigella*, *Lactobacillus*, *Parabacteroides*, *Clostridium_sensu_stricto_1*, *Prevotellaceae_NK3B31_group*, and *Prevotella*. The top 10 predominant bacteria at day 70 of age were mainly involved in *Prevotellaceae_UCG-003*, *Prevotellaceae_NK3B31_group*, *Terrisporobacter*, *Alloprevotella*, *Lactobacillus*, *Faecalibacterium*, *Treponema*, *Rikenellaceae_RC9_gut_group*, *Clostridium_sensu_stricto_1*, and *Prevotella* (Figure 5B).

**FIGURE 6:**
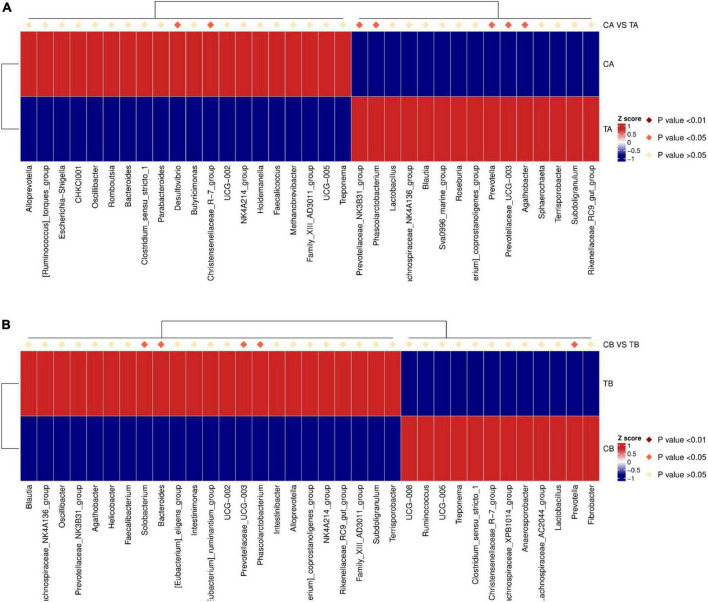
Top 35 species abundance clustering with ComplexHeatmap (genus level) for intestinal microbiota of piglets weaned on day 14 of age (14W) and day 28 of age (28W) at different sampling timepoints [day 28 of age **(A)**; day 70 of age **(B)**]. Underside represents the species annotation information. Upside represents the contrast between the groups. The corresponding value of the heat map is the Z value of the relative abundance of species in each row after the normalization treatment. CA group was defined as the sample from piglets in 28W group at the timepoint of day 28 of age. TA group was defined as the sample from piglets in 14W group at the timepoint of day 28 of age **(A)**. CB group was defined as the sample from piglets in 28W group at the timepoint of day 70 of age. TB group was defined as the sample from piglets in 14W group at the timepoint of day 70 of age **(B)**.

Heatmap annotations are important components of a heatmap that show additional information associated with rows of columns. ComplexHeatmap provides very flexible support for setting annotations and defining new annotation graphics. On day 28 of age (Figure 6A), in comparison with TA group (14W-28), the richness of *Desulfovibrio* (*P* = 0.015) and *Christensenellaceae_R-7_group* (*P* = 0.044) was significantly upregulated and that of *Prevotellaceae_NK3B31_group* (*P* = 0.045), *Phascolarctobacterium* (*P* = 0.033), *Prevotella* (*P* = 0.045), *Prevotellaceae_UCG-003* (*P* = 0.013), and *Agathobacter* (*P* = 0.034) was significantly downregulated in the group of CA (28W-28). On day 70 of age (Figure 6B), the richness of *Solobacterium* (*P* = 0.049), *Bacteroides* (*P* = 0.015), *Prevotellaceae_UCG-003* (*P* = 0.033), and *Phascolarctobacterium* (*P* = 0.046) was significantly upregulated and that of *Prevotella* (*P* = 0.022) was significantly downregulated in the group of TB (14W-70) in comparison with those in the CB group (28W-70).

## Discussion

The lengths of suckling period have been reported to affect the growth performance of piglets during post-weaning (Massacci et al., 2018; Faccin et al., 2020a,b). Collins et al. (2010) reported that piglets receiving 21-day suckling period had two times higher ADFI and weight gain than those receiving 13-day suckling period during the 11 days post-weaning. Faccin et al. (2020b) noted that the impairment of body weight and feed efficiency induced by weaning was ameliorated by prolonging the suckling period. Dunshea et al. (2002) found that piglets weaned on day 25 of age had a higher growth rate than those weaned on day 17 of age during 3 weeks post-weaning. In this study, we have also observed the worst growth performance of piglets in the ultra-early weaning group. Some studies attributed this growth performance impairment to the reduction in voluntary feed intake (Van der Meulen et al., 2010; Ming et al., 2021). Main et al. (2004) noted that prolonging suckling period would increase the acceptability of solid feed during post-weaning. The reduction in ADFI was also observed in this study; therefore, we speculated that the impairment of growth performance induced by shortening suckling period was related to the decrease in feed intake. Additionally, we observed that the growth performance impairment continued to day 70 of age, which means the growth impairment effect persisted throughout the overall experimental periods. Interactions between time and treatment were also observed for body weight, ADG, and growth rate in this study, which indicated that the impairment of growth performance was aggravated with the passage of time. Conversely, Partanen et al. (2007) reported that piglets allowed to suckle until day 36 of age had a heavier body weight and a growth rate than those weaned on day 26 of age; however, the difference in body weight was diminished on day 49 of age. We have not observed the short-term growth impairment effect as similar to the above studies, which was probably due to the difference in genetic background. As the genetic background plays a key role in affecting individual differences (Alexander et al., 2008), this study was the first time to evaluate the effects of shortening suckling period on growth performance of piglets with the same genetic background. We considered that ultra-early weaning had negative effects on the growth performance of piglets, which was partially attributed to the reduction in voluntary feed intake.

Additionally, the composition of intestinal microbiota plays a close relationship with growth performance. Thousands of organism communities constitute the gut microbiome. The large diversity of the microbiota contributes to the development and metabolic needs of the host. Piglets have not established stable intestinal microbiota during the early life. It is reported that shortening suckling period of piglets would decrease the resistance to the pathogenic *Escherichia coli*, which was manifested in severe diarrhea, body weight reduction, and pathogen shedding (Wellock et al., 2008; McLamb et al., 2013). In addition, some studies indicated that shortening suckling periods of piglets would lead to the enrichment of harmful bacteria in the feces (Leliveld et al., 2013; Xu et al., 2014). Therefore, the duration of suckling period, also named weaning age, plays an important role in regulating the intestinal microbiota (Yang et al., 2018). Breast milk oligosaccharides have been shown to stimulate the growth of bifidobacteria and lactobacilli in the intestine of infants (Villares, 2008). Moreover, breast milk also presents various immunostimulatory factors, anti-inflammatory factors, and antimicrobial substances (Blewett et al., 2008). Therefore, the components presented in the milk will affect the intestinal microbiota to some extent (Schack-Nielsen and Michaelsen, 2007). The Chao1 index and Ace index are two indicators used to estimate species richness of intestinal microbiota. The Shannon and Simpson diversity values are indices used to estimate the microbial diversity in the samples. The bacterial community was analyzed following high-throughput pyrosequencing of 16S rRNA genes, and we found that ultra-early weaning led to a reduction in intestinal microbiota richness; however, this reduction effect was only observed on day 28 of age, but not on day 70 of age, which indicated that ultra-early weaning temporarily affected the richness of intestinal microbiota in piglets. This result was affirmed by the studies of Massacci et al. (2018) and Holman et al. (2021). Similarly, Massacci et al. (2018) observed a higher alpha-diversity in intestinal microbiota caused by prolonging suckling periods. Animals with richer microbiota are capable of increasing the resistance to enteric diseases during post-weaning period and possibly providing a competitive advantage to piglets (Massacci et al., 2018). In this study, the *Firmicutes* and *Bacteroidetes* were the dominant phyla in both timepoints, which was affirmed by the study of Yang et al. (2018). In addition, on day 28 of age, ultra-early weaning led to an increase in bacteria related to the production of short-chain fatty acids (SCFA), such as *Agathobacter* (Horvath et al., 2021), *Prevotellaceae_NK3B31_group* (Shang et al., 2021), *Prevotella* (Yang et al., 2018), and *Phascolarctobacterium* (Yang et al., 2018). The production of SCFA is important in energy homeostasis (Schwiertz et al., 2010; Blaut, 2015). In addition, ultra-early weaning led to an increase in intestinal *Prevotellaceae_UCG-003*, which is closely related to polysaccharide, protein, energy, and vitamin metabolism (Cui et al., 2022), and a decrease in *Desulfovibrio* and *Christensenellaceae_R.7_group*, which are the bacteria involved in inducing bowel disease (Marini et al., 2002; Mancabelli et al., 2017). On day 70 of age, ultra-early weaning led to an increase in *Bacteroides* (Yang et al., 2018) and *Phascolarctobacterium* (Yang et al., 2018), which are related to the production of SCFA, as well as *Prevotellaceae_UCG-003*, which is related to polysaccharide, protein, energy, and vitamin metabolism (Cui et al., 2022), but a decrease in *Prevotella*, which is involved in infections (Giri and Mangalam, 2019). Therefore, ultra-early weaning seems to increase beneficial bacteria and decrease harmful bacteria in the intestine and thus benefit establishing a healthy intestinal microbiota during the early life of piglets, which was affirmed by the study of Smith et al. (2008); shortening suckling period would decrease the risk of the vertical transmission of pathogens from sows to offspring.

On the contrary, the intestinal microbiota compositions are closely related to the immune status of the host. Most bacterial species are capable of inducing a strong host immunity response (Macpherson et al., 2005). Immunoglobulin is one of the important components in the immunity system, which mainly exists in the serum and intestinal mucosa. In this study, we investigated the effects of ultra-early weaning on the contents of serum IgA, IgG, and IgM. We found that ultra-early weaning had positive effects on the immunological parameters on day 28 of age, but not on day 70 of age. Altering the lengths of suckling period has been demonstrated to affect the immune status of piglets (Cao et al., 2022). Salak-Johnson and Webb (2018) noted that piglets allowed 28-day suckling period had higher serum immunoglobulin contents than those weaned on day 14 of age. Some studies noted that shortening suckling period was capable of decreasing the serum IgA contents (Levast et al., 2010; Smith et al., 2010; Cao et al., 2022). Levast et al. (2010) demonstrated that the reduction in serum IgA levels induced by shortening suckling period was closely related to the intestinal environment. In this study, on day 28 of age, we found that the content of IgA was positively correlated with the abundance of *Prevotellaceae_NK3B31_group* and *Agathobacter*, that of IgG was positively correlated with the abundance of *Prevotellaceae_UCG.003*, and that of IgM was positively correlated with the abundance of *Prevotella*. However, no bacteria were positively correlated with the immunological parameters on day 70 of age. Therefore, we considered that the variation of intestinal microbiota composition induced by shortening suckling period would activate the immune system during the early life of piglets, which was manifested in the increase in serum immunoglobulin levels; however, this activation effect was not long term, but temporary.

Serum biochemical parameters including albumin, globulin, and total protein can be used as an indicator to indicate the situation of protein synthesis and nutritional status *in vivo* (Park and Kim, 2019). In this study, we observed low albumin and total protein contents as well as albumin-to-globulin ratio in the group of 14W in comparison with those in the group of 28W on day 28 of age, but not on day 70 of age. Similarly, Tao et al. (2016) and Hohenshell et al. (2000) reported that the effects of shortening suckling period on serum biochemical indicators were temporary and could be corrected to normal levels within some time post-weaning. This indicated that shortening suckling period will cause a malnutrition status for piglets, which was probably the reason for growth retardation as observed in this study. The malnutrition of piglets during early life may affect the development of other organs, which allows a severe challenge for the further growth of piglets.

## Conclusion

This study demonstrated that shortening suckling period of piglets had a long-term effect on the impairment of growth performance, whereas it had a short-term effect on the increase in serum immunoglobulin parameters as well as the decrease in serum biochemical indicators and intestinal species abundance. Additionally, we observed that ultra-early weaning was capable of increasing the intestinal beneficial bacteria, but decreasing the pathogenic bacteria. Therefore, we considered that ultra-early weaning had positive effects on the immunity status and intestinal microbiota composition in piglets, but negative effects on the growth performance, nutritional status, and intestinal microbiota abundance. In the aspect of growth performance, the lower the weaning weight, the longer the time needed to reach marketing weight will be, which inevitably impaired profitability. However, we did observe an optimization of intestinal microbiota composition in piglets caused by shortening suckling period. Combining ultra-early weaning with other nutritional strategies may be an appropriate strategy to improve the overall post-weaning performance in piglets.

## Data availability statement

The raw data supporting the conclusions of this article will be made available by the authors, without undue reservation.

## Ethics statement

Experimental protocol and the process were approved and supervised by the Animal Care and Use Committee of Jinzhou Medical University (Jinzhou, China).

## Author contributions

DD and CL were involved in writing – original draft, investigation, and writing – review and editing. SL, XF, and WX were involved in formal analysis and investigation. YC and DL were involved in conceptualization, methodology, supervision, and writing – review and editing. All authors contributed to the article and approved the submitted version.

## References

[B1] Alexander L. S. Qu A. Cutler S. A. Mahajan A. Lonergan S. M. Rothschild M. F. (2008). Response to dietary phosphorus deficiency is affected by genetic background in growing pigs. *J. Anim. Sci.* 86 2585–2595. 10.2527/jas.2007-0692 18502882

[B2] Blaut M. (2015). Gut microbiota and energy balance: Role in obesity. *Proc. Nutr. Soc.* 74 227–234. 10.1017/S0029665114001700 25518735

[B3] Blewett H. J. H. Cicalo M. C. Holland C. D. Field C. J. (2008). The immunological components of human milk. *Adv. Food Nutr. Res.* 54 45–80. 10.1016/S1043-4526(07)00002-2 18291304

[B4] Buchet A. Belloc C. Leblanc-Maridor M. Merlot E. (2017). Effects of age and weaning conditions on blood indicators of oxidative status in pigs. *PLoS One* 12:e0178487. 10.1371/journal.pone.0178487 28542567 PMC5443573

[B5] Cao S. Hou L. Sun L. Gao J. Gao K. Yang X. (2022). Intestinal morphology and immune profiles are altered in piglets by early-weaning. *Int. Immunopharmacol.* 105:108520. 10.1016/j.intimp.2022.108520 35063748

[B6] Champy M. F. Selloum M. Zeitler V. Caradec C. Jung B. Rousseau S. (2008). Genetic background determines metabolic phenotypes in the mouse. *Mamm. Genome* 19 318–331. 10.1007/s00335-008-9107-z 18392653

[B7] Collins C. L. Leury B. J. Dunshea F. R. (2010). Early weaning has minimal effects on lifetime growth performance and body composition of pigs. *Anim. Prod. Sci.* 50 79–87. 10.1071/AN09059

[B8] Cui Y. Liu H. Gao Z. Xu J. Liu B. Guo M. (2022). Whole-Plant Corn Silage Improves Rumen Fermentation and Growth Performance of Beef Cattle by Altering Rumen Microbiota. *Appl. Microbiol. Biotechnol.* 106 4187–4198. 10.1007/s00253-022-11956-5 35604439

[B9] Dunshea F. R. Kerton D. K. Cranwell P. D. Campbell R. G. Mullan B. P. King R. H. (2002). Interactions between weaning age, weaning weight, sex, and enzyme supplementation on growth performance of pigs. *Aust. J. Agric. Res.* 53 939–945. 10.1071/AR01197

[B10] EFSA Panel on Animal Health and Welfare (2022). Welfare of pigs on farm. *EFSA J.* 20:e07421. 10.2903/j.efsa.2022.7421 36034323 PMC9405538

[B11] Faccin J. E. Tokach M. D. Allerson M. W. Woodworth J. C. DeRouchey J. M. Dritz S. S. (2020a). Relationship between weaning age and antibiotic usage on pig growth performance and mortality. *J. Anim. Sci.* 98:skaa363. 10.1093/jas/skaa363 33188416 PMC7755175

[B12] Faccin J. E. Laskoski F. Hernig L. F. Kummer R. Lima G. F. Orlando U. A. (2020b). Impact of increasing weaning age on pig performance and belly nosing prevalence in a commercial multisite production system. *J. Anim. Sci.* 98:skaa031. 10.1093/jas/skaa031 32034395 PMC7183176

[B13] Federation of Animal Science Societies (2010). *Guide for the care and use of agriculture animals in research and teaching.* Champaign: FASS.

[B14] Giri S. Mangalam A. K. (2019). “The Gut Microbiome and Metabolome in Multiple Sclerosis,” in *Microbiome and metabolome in diagnosis, therapy, and other strategic applications*, eds Faintuch J. Faintuch S. (London: Elsevier), 333–340.

[B15] Hohenshell L. M. Cunnick J. E. Ford S. P. Kattesh H. G. Zimmerman D. R. Wilson M. E. (2000). Few differences found between early-and late-weaned pigs raised in the same environment. *J. Anim. Sci.* 78 38–49. 10.2527/2000.78138x 10682801

[B16] Holman D. B. Gzyl K. E. Mou K. T. Allen H. K. (2021). Weaning age and its effect on the development of the swine gut microbiome and resistome. *mSystems* 6 e00682–e00721. 10.1128/mSystems.00682-21 34812652 PMC8609972

[B17] Horvath A. Bausys A. Sabaliauskaite R. Stratilatovas E. Jarmalaite S. Schuetz B. (2021). Distal gastrectomy with Billroth II reconstruction is associated with oralization of gut microbiome and intestinal inflammation: A proof-of-concept study. *Ann. Surg. Oncol.* 28 1198–1208. 10.1245/s10434-020-08678-1 32504369 PMC7801296

[B18] Huting A. M. Wellock I. Tuer S. Kyriazakis I. (2019). Weaning age and post-weaning nursery feeding regime are important in improving the performance of lightweight pigs. *J. Anim. Sci.* 97 4834–4844. 10.1093/jas/skz337 31679028 PMC6915233

[B19] Isaacson R. Kim H. B. (2012). The intestinal microbiome of the pig. *Anim. Health Res. Rev.* 13 100–109. 10.1017/S1466252312000084 22853934

[B20] Leliveld L. M. C. Riemensperger A. V. Gardiner G. E. O’Doherty J. V. Lynch P. B. Lawlor P. G. (2013). Effect of weaning age and postweaning feeding programme on the growth performance of pigs to 10 weeks of age. *Livest. Sci.* 157 225–233. 10.1016/j.livsci.2013.06.030

[B21] Levast B. de Monte M. Chevaleyre C. Melo S. Berri M. Mangin F. (2010). Ultra-early weaning in piglets results in low serum IgA concentration and IL17 mRNA expression. *Vet. Immunol. Immunop.* 137 261–268. 10.1016/j.vetimm.2010.06.004 20591504

[B22] Mabry J. W. Culbertson M. S. Reeves D. (1996). Effects of lactation length on weaning-to-first-service interval, first-service farrowing rate, and subsequent litter size. *Swine Health Prod.* 4 185–188.

[B23] Macpherson A. J. Geuking M. B. McCoy K. D. (2005). Immune responses that adapt the intestinal mucosa to commensal intestinal bacteria. *Immunology* 115 153–162. 10.1111/j.1365-2567.2005.02159.x 15885120 PMC1782138

[B24] Main R. G. Dritz S. S. Tokach M. D. Goodband R. D. Nelssen J. L. (2004). Increasing weaning age improves pig performance in a multisite production system. *J. Anim. Sci.* 82 1499–1507. 10.2527/2004.8251499x 15144093

[B25] Main R. G. Dritz S. S. Tokach M. D. Goodband R. D. Nelssen J. L. (2005). Effects of weaning age on growing-pig costs and revenue in a multi-site production system. *J. Swine Health Prod.* 13 189–197.

[B26] Mancabelli L. Milani C. Lugli G. A. Turroni F. Cocconi D. van Sinderen D. (2017). Identification of universal gut microbial biomarkers of common human intestinal diseases by meta-analysis. *FEMS Microbiol. Ecol.* 93:fix153. 10.1093/femsec/fix153 29126267

[B27] Marini R. P. Otto G. Erdman S. Palley L. Gox J. G. (2002). “Biology and Diseases of Ferrets,” in *Laboratory Animal Medicine (Second Edition)*, eds Fox J. G. Anderson L. C. Loew F. M. Quimby F. W. (Cambridge, MA: Academic Press), 483–517. 10.1016/B978-012263951-7/50016-8

[B28] Massacci F. Berri E. M. Lemonnier G. Jardet D. Blanc F. Revilla Sanchez M. (2018). “Impact of weaning age on the gut microbiota composition in piglets,” in *69*^th^* Annual Meeting of the European Federation of Animal Science.* (Wageningen: Wageningen Academic Publishers), 704.

[B29] McLamb B. L. Gibson A. J. Overman E. L. Stahl C. Moeser A. J. (2013). Early weaning stress in pigs impairs innate mucosal immune responses to enterotoxigenic E. coli challenge and exacerbates intestinal injury and clinical disease. *PLoS One* 8:e59838. 10.1371/journal.pone.0059838 23637741 PMC3634819

[B30] Ming D. Wang W. Huang C. Wang Z. Shi C. Ding J. (2021). Effects of weaning age at 21 and 28 days on growth performance, intestinal morphology and redox status in piglets. *Animals* 11:2169. 10.3390/ani11082169 34438627 PMC8388437

[B31] National Research Council [NRC] (2012). *Nutrient requirements of swine, 11th revised edition.* Washington, DC: National Academies Press.

[B32] Nuntapaitoon M. (2022). “Colostrum and Milk in Sow,” in *The Milk Protein: New Research Approaches*, ed. Chaiyabutr N. (Norderstedt: Books on Demand), 93.

[B33] Park J. H. Kim I. H. (2019). The effects of betaine supplementation in diets containing different levels of crude protein and methionine on the growth performance, blood components, total tract nutrient digestibility, excreta noxious gas emission, and meat quality of the broiler chickens. *Poult. Sci.* 98 6808–6815. 10.3382/ps/pez412 31347674 PMC8913992

[B34] Partanen K. Siljander-Rasi H. Pentikäinen J. Pelkonen S. Fossi M. (2007). Effects of weaning age and formic acid-based feed additives on pigs from weaning to slaughter. *Arch. Anim. Nutr.* 61 336–356. 10.1080/17450390701556866 18030917

[B35] Salak-Johnson J. L. Webb S. R. (2018). Short-and long-term effects of weaning age on pig innate immune status. *J. Anim. Sci.* 8:137. 10.4236/ojas.2018.82010

[B36] Schack-Nielsen L. Michaelsen K. F. (2007). Advances in our understanding of the biology of human milk and its effects on the offspring. *J. Nutr.* 137 503S–510S. 10.1093/jn/137.2.503S 17237337

[B37] Schwiertz A. Taras D. Schäfer K. Beijer S. Bos N. A. Donus C. (2010). Microbiota and SCFA in lean and overweight healthy subjects. *Obesity* 18 190–195. 10.1038/oby.2009.167 19498350

[B38] Shang Q. Liu S. Liu H. Mahfuz S. Piao X. (2021). Impact of sugar beet pulp and wheat bran on serum biochemical profile, inflammatory responses and gut microbiota in sows during late gestation and lactation. *J. Anim. Sci. Biotechnol.* 12 1–14. 10.1186/s40104-021-00573-3 33879267 PMC8059298

[B39] Smith A. L. Stalder K. J. Serenius T. V. Baas T. J. Mabry J. W. (2008). Effect of weaning age on nursery pig and sow reproductive performance. *J. Swine Health Prod.* 16 131–137.

[B40] Smith F. Clark J. E. Overman B. L. Tozel C. C. Huang J. H. Rivier J. E. (2010). Early weaning stress impairs development of mucosal barrier function in the porcine intestine. *Am. J. Physiol. Gastrointest.* 298 G352–G363. 10.1152/ajpgi.00081.2009 19926814 PMC2838512

[B41] Spencer J. D. Boyd R. D. Cabrera R. Allee G. L. (2003). Early weaning to reduce tissue mobilization in lactating sows and milk supplementation to enhance pig weaning weight during extreme heat stress. *J. Anim. Sci.* 81 2041–2052. 10.2527/2003.8182041x 12926786

[B42] Tao X. Xu Z. Men X. (2016). Transient effects of weaning on the health of newly weaning piglets. *Czech J. Anim. Sci.* 61 82–90. 10.17221/8731-CJAS

[B43] Van der Meulen J. Koopmans S. J. Dekker R. A. Hoogendoorn A. (2010). Increasing weaning age of piglets from 4 to 7 weeks reduces stress, increases post-weaning feed intake but does not improve intestinal functionality. *Animal* 4 1653–1661. 10.1017/S1751731110001011 22445118

[B44] Villares J. M. (2008). Prebiotics in Infant Formulae. Could we modify the immune response?. *An. Pediatr.* 68 286–294. 10.1157/13116712 18358143

[B45] Wellock I. J. Fortomaris P. D. Houdijk J. G. M. Kyriazakis I. (2008). Effects of dietary protein supply, weaning age and experimental enterotoxigenic *Escherichia coli* infection on newly weaned pigs: Health. *Animal* 2 834–842. 10.1017/S1751731108002048 22443662

[B46] Xu J. Xu C. Chen X. Cai X. Yang S. Sheng Y. (2014). Regulation of an antioxidant blend on intestinal redox status and major microbiota in early weaned piglets. *Nutrition* 30 584–589. 10.1016/j.nut.2013.10.018 24698350

[B47] Xue J. Dial G. D. Marsh W. E. Lucia T. (1997). Association between lactation length and sow reproductive performance and longevity. *J. Am. Vet. Med. Assoc.* 210 935–938. 9096723

[B48] Yang H. Xiao Y. Wang J. Xiang Y. Gong Y. Wen X. (2018). Core gut microbiota in Jinhua pigs and its correlation with strain, farm and weaning age. *J. Microbiol.* 56 346–355. 10.1007/s12275-018-7486-8 29721832

